# Effect of Age on the Touchscreen Manipulation Ability of Community-Dwelling Adults

**DOI:** 10.3390/ijerph18042094

**Published:** 2021-02-21

**Authors:** Michal Elboim-Gabyzon, Patrice L. Weiss, Alexandra Danial-Saad

**Affiliations:** 1 Department of Physical Therapy, Faculty of Social Welfare & Health Sciences, University of Haifa, 3498838 Haifa, Israel; 2 Department of Occupational Therapy, Faculty of Social Welfare & Health Sciences, University of Haifa, 3498838 Haifa, Israel; plweiss@gmail.com (P.L.W.); saadalexandra@gmail.com (A.D.-S.); 3 The Arab Academic College for Education in Israel, University of Haifa, 3498838 Haifa, Israel

**Keywords:** hand function, touchscreen manipulation skills, disability, assessment tool, usability, older adults, assistive technology, low cost, outcome measures

## Abstract

Assessment of touchscreen manipulation skills is essential for determining the abilities of older individuals and the extent to which they may benefit from this technology as a means to enhance participation, self-esteem, and quality of life. The aim of this study was to compare the touchscreen manipulation ability between community-dwelling older adults and middle-aged adults using a newly developed Touchscreen Assessment Tool (TATOO) and to determine the usability of this instrument. Convenience samples of two age groups were considered, one including 28 independent community-living older adults aged 81.9 ± 4.2 years with intact or corrected vision and with the abilities to walk independently with or without a walking aid and to understand and follow simple commands, and the other including 25 healthy middle-age adults aged 53.4 ± 5.9 years. The usability assessment was conducted during a single session using the System Usability Scale (SUS). Older adults demonstrated poorer touchscreen skills compared to middle-aged adults. Previous experience in manipulating a smartphone by the older adults did not affect their performance. The SUS results indicated good usability of the TATOO by both age groups. The TATOO shows promise as a user-friendly tool for assessing the specific skills needed to operate touchscreens. The outcomes of this study support the suitability of touchscreen devices and applications as well as the need for adapted accessibility for older adults. Researchers and clinicians will benefit from the availability of a rapid, low-cost, and objective tool to assess the skills required for touchscreen use.

## 1. Introduction

Hand function is an important component of one’s ability to lead an independent and active life within a community. This is particularly relevant among the rapidly growing population of individuals over the age of 70 years [1]. Loss of manual hand functioning in this age group often results from natural age-related deterioration in the musculoskeletal and nervous systems [2]. The impact of these changes may lead to difficulties in performing basic and instrumental activities of daily life (Basic Activities of Daily Living (BADL) and instrumental Activities of Daily Living (IADL), respectively) [3].

The approach recommended by the International Classification of Functioning, Disability, and Health (ICF) [4] for evaluating hand function in older adults focuses on assessments targeting two broad domains. The first relates to impairments in the sensorimotor system including reduced muscle strength, reduced tactile sensibility, and slower motor responses [3,5,6]. The second focuses on the skills needed to perform BADL and IADL, which impact one’s independence and participation in daily occupations. The skills in the latter domain have traditionally been assessed by observing the performance of activities such as tying shoelaces, buttoning buttons, selecting coins from a wallet, or pouring milk into a cup [5,6,7]. 

In modern society, participation in daily occupations often entails access to touchscreen devices such as tablets and smartphones due to their convenient and user-friendly interfaces, portability, affordability, regardless of the user’s age [8,9]. These devices can be used to operate multiple services such as banking services, access to health care, home automation [8]. 

Insufficient attention has been paid to the clinical assessment of the hand function skills needed to operate technology-based devices such as tablets and smartphones, especially the fine motor skills needed to interact with a touchscreen interface. Such data are of particular importance to determine the extent to which older adults are able to cope with the use of such devices that support an active lifestyle by increasing physical exercise, decreasing social isolation, supporting social communication, and increasing participation in meaningful occupations [10,11]. Usage of these devices may enhance the cognitive and physical performance of community-dwelling older adults and provide monitoring technologies in order to increase their independence, autonomy, quality of life, security [12,13,14] and improve their medical management [15,16]. 

Previous studies on the performance of older adults in using touchscreens have focused primarily on ergonomic, design, or usability aspects, technology acceptance, usage behavior patterns [8,14,17,18,19,20], and cognitive assessment [21]. Their use as a tool to assess hand performance during goal-oriented touchscreen tasks has been reported with less frequency, but there is evidence that older adults demonstrate poorer performance (e.g., decreased accuracy) in tablet-based puzzles compared to younger adults [22] and that, when tracing geometric shapes with a single finger on the touchscreen of a small mobile device, they perform more slowly and make more errors [23]. Thus, there is a need to further assess the unique hand skills needed to operate touchscreen devices.

The Touchscreen Assessment Tool (TATOO) is a software application developed by Danial-Saad and Chiari (2018) [24] to assess touchscreen manipulation skills. It consists of six tasks providing information on the performance of the functional components required to use a touchscreen effectively (i.e., tapping, pinching, and dragging). The outcomes of each task are summarized by numeric and graphic reports (see additional detail below [24]). 

The current study was designed to compare the touchscreen manipulation ability between community-dwelling older adults (>75 years) and middle-aged adults (45–65 years) and to determine the usability of the TATOO as reported by the participants.

## 2. Materials and Methods

Twenty-eight older adults, aged 75 years and older, were recruited via convenience sampling at community centers, and 25 middle-age adults, aged 45–65 years were recruited via social media. This sample size was based on data from a previous study [25] for use with the T-test for equal-size groups, at 5% significance level and 85% power using SAS software version 9.4 (SAS, Cary, NC, USA). All participants were able to understand and follow simple commands, lived independently in the community, and were able to walk on their own with or without a walking aid. Participants were excluded from the study if they had moderate to severe balance or gait impairments, chronic medical conditions that could affect hand function (e.g., neurological, orthopedic, or cardiopulmonary diseases), severe pain that adversely affected the performance of functional daily activities, or uncorrected vision or hearing impairments.

The study was approved by the Ethical Review Board at the University of Haifa. All participants provided written informed consent before participation, after a detailed explanation of the study objectives and design.

### 2.1. Instruments

Demographic characteristics: An in-person structured interview was used to collect personal characteristics (e.g., age, gender), health status, and answers to questions regarding level of independence in BAD and IADL. Participants were also asked about their experience with operating technological devices, including hand dominance. 

TATOO software application: The TATOO [24,26] was developed to evaluate the ability to manipulate a touchscreen. It consists of six tasks: (1) touch the entire screen; (2) touch all corners; (3) double tapping; (4) drag in all directions; (5) drag along straight horizontal paths of varying lengths; (6) pinching skill (see details in Danial-Saad et al. [26]). The quantitative task outcomes provide data on the performance of the functional components required to use a touchscreen effectively (i.e., tapping, swiping, pinching, and dragging) including (1) temporal variables (i.e., reaction time and task duration) and (2) performance variables (i.e., number of fingers used to operate each skill and movement accuracy, assessed using indices such as the number of taps completed successfully, the location of the first tap, and the number of drag attempts successfully completed or not completed). Two additional outcome measures—‘flight’ time and ‘touch’ time—were collected for tasks 2–6. ‘Flight’ time is defined as the time during which the hand is not in contact with the surface of the tablet, and ‘touch’ time is defined as the time during which the hand is in contact with the surface. 

Usability Assessment: The System Usability Scale (SUS) [27] was used to obtain the participant’s perception regarding the usability of the TATOO. The scale includes 10 items covering three facets of usability: effectiveness, efficiency, and satisfaction. The items were rated on a 5-point Likert scale, in which “1” indicated complete disagreement, and “5” indicated complete agreement to 10 positive or negative statements. The total score was calculated in two steps that entailed transformation and reversion to a 4-point scale and calculation of the total score by summing the scores of all 10 items and multiplying by 2.5. The tool score ranges between 0 and 100 [28,29]. Scores equal to or above 68 are considered to be “good’’, and scores below 50 are considered to be ‘‘very weak’’ [12,30]. Internal consistency of the SUS was found to be high (α = 0.91), and concurrent validity was found to be moderate (r = 0.81, *p* < 0.001) [31]. 

### 2.2. Procedure

Each participant underwent a series of assessments conducted in a fixed order during a single 45 min session. The assessments were: (1) an in-person structured interview recording personal characteristics and data regarding experience with operating a smartphone and frequency of use; (2) touchscreen manipulation ability: beginning with the dominant hand, the subjects performed six TATOO tasks while sitting comfortably in front of the touchscreen placed on a stable table. Each task was repeated once following detailed verbal and modeling instructions. The duration of the entire test was approximately 15 min. Subjects habitually using glasses were requested to use them while performing the tasks; (3) TATOO usability: following completion of the TATOO test, the user was requested to complete the SUS.

### 2.3. Statistical Analysis

Descriptive statistics was applied to the data regarding the personal characteristics and experience with operating technologic devices, the six TATOO tasks, and the usability scores. A two-way, mixed-model, repeated-measures analysis of variance (ANOVA) was performed to examine the effect of group and hand on the outcome variables. Univariate analyses were performed using Chi-square, Wilcoxon two-sample, or Kruskal–Wallis tests. Benjamini and Hochberg’s [32] methods were used for adjusting pairwise comparisons; *p*-values ≤ 0.05 were considered statistically significant. All statistical analyses were performed by the R language and environment for statistical computing and graphics (R Foundation for Statistical Computing, version 3.0.1, http://www.R-project.org (accessed on 13 January 2021)).

## 3. Results

### 3.1. Participants 

Twenty-eight community-dwelling older adults with a mean age of 81.9 ± 4.2 years (range, 75–90 years) and 25 middle-aged individuals with a mean age of 53.4 ± 5.9 years (range, 46–63 years) participated in this study (see Table 1). All the participants reported being independent in both BADL and IADL. Participants were all right-hand dominant.

### 3.2. TATOO Performance Results 

The results of the TATOO tasks demonstrated poorer performance by the older adult group versus the middle-aged group in both the temporal variables (e.g., longer reaction times, longer duration times, longer touch and flight times) and the performance variables (e.g., greater accuracy, larger numbers of drags completed and of touches outside the target), as shown in Table 2. These differences were significant for 80% of the variables (28 out of 35, with *p* values ranging from <0.0001 to 0.04). Of the six TATOO tasks, two were considered to be simple, since they entailed direct, single-step movements and required less accuracy (“touch the entire screen” and “touch all corners”). The other four tasks were more complex, since they entailed indirect, multi-step movements with greater accuracy (“double tapping”, “drag in all directions”, “drag along straight horizontal paths of varying lengths”, and “pinching skills”). 

As indicated in Table 2, there were very few significant differences (8 out of 35 variables) when performing the tasks with the right or the left hand. No significant interactions between hand and group were found, except for the two variables in Task 1 and one variable in Task 4.

A significant difference (*p* = 0.001) was found between older adults (57.1%) compared to middle-aged adults (100%) in terms of smartphone ownership. Regarding the frequency of usage, all the middle-aged adults reported their use of a smartphone several times a day, while only 57.1% of the older adults indicated using the smartphone a few times a day, and the rest indicated not using it at all. The most common use of a smartphone by the older adults was for communication (phoning) and photography. None of the older adults reported using a smartphone for shopping. No significant difference was found for the performance of selected temporal and accuracy TATOO outcome measures (those that were found to show significant differences for age) between older adults who owned a smartphone or who used a smartphone a few times a day and those who did not. 

### 3.3. Usability Data of the TATOO Application 

Usability of the TATOO application was reported to be good by both the older and the middle-age group, as determined by the SUS scores (mean ± standard deviation 80.19. ± 17.91 and 82.80 ± 13.66, respectively) with no significant difference between them. Detailed results of the SUS items are presented in Table 3. 

## 4. Discussion

The primary aim of this study was to assess the ability of ambulatory community-dwelling older adults to manipulate a touchscreen in comparison to middle-aged adults. The results showed that the older adult participants demonstrated significantly poorer performance in many of the temporal and accuracy measures, including longer reaction and task duration times, more taps to achieve the desired goal, and more frequent taps outside the target zones. These results support those of prior studies indicating that older adults are less proficient in using computer input devices such as a mouse or a touchpad [25,33]. These differences appear to be linked to an age-related decline in motor skills, since opportunities for practice as provided by prior smartphone ownership and usage did not affect the current between-group differences. 

Age-related differences were noted in the performance of the tasks which involved complex movements such as those that entailed rapid sequences and greater accuracy. Significant group differences were found in the performance of the more motorically challenging actions such as those required in the “double tapping” task [34]; the older adult participants required significantly more time to complete this task, needed more attempts to perform it correctly, and exhibited more taps falling outside the target zone. These results are consistent with those of previous studies in which the performance of older adults was related to task difficulty. For example, elderly individuals had greater difficulty in pointing tasks or clicking, double-clicking, and dragging tasks [18,25,34]. 

Our results also revealed that both ‘flight’ time and ‘touch’ time variables were significantly longer in the older adult group for all six TATOO tasks. The longer times were likely due to overall delays in motor performance in the elderly participants [35], likely resulting from age-related central and peripheral neuromuscular deterioration reflected in longer visual processing time, less effective integration of visuospatial-sensory input and motor output, slower movement speed, less efficient planning of movement paths, greater difficulty in target selection, and inefficient acceleration–deceleration patterns with an increased number of sub-movements [36]. 

Data from assessments such as those obtained with the TATOO are designed to enable clinicians and touchscreen software developers to provide tailor-made recommendations for touchscreen usage and development that will accommodate each user’s abilities relative to the specific device they use (e.g., active area size, touch sensitivity [37]) as well as those to specific functions (e.g., dwell time, accuracy, speed). A key element for the clinical implementation of effective assessment tools relates to their usability for a target population. The results of the SUS indicated that participants in both groups reported good usability ratings for the TATOO. This finding supports its suitability for use in assessing touchscreen manipulation ability among older adults in relation to three facets of usability: effectiveness, efficiency, and satisfaction [38].

The present results revealed very few significant differences between dominant and nondominant hands for tablet tasks. It may be that the manipulation of a touchscreen does not require the level of dexterity of activities such as handwriting or buttoning. It is interesting to note that previous studies on older adults have demonstrated a similarity of hand performance in a variety of motor tasks with a tendency for older people to use both hands frequently (equalization of hand performance) during everyday activities [39,40]. Previous studies suggested that the phenomenon of age-related equalization between hand performance abilities may be that age-related deterioration in hand functioning appears to be asymmetrical, with the decline of the dominant hand being greater than that of the non-dominant hand [39]. Finally, hand preference may be dependent on task difficulty [41], although a consensus has yet to be determined on this issue. While the present study indicates equalization between hand performance, it does not indicate whether this was due to task difficulty or was related to a decreased ability of the dominant hand. Further studies are required to clarify the relation between age-related changes in hand dominance and touchscreen manipulation abilities in tasks of varying complexity.

Given that the study population included community-living older adults, the results cannot be generalized to frail elderly or to those with hand impairments (e.g., due to osteoarthritis). Other factors such as cognitive status and gender were not included in the analyses. Since the current study did not assess BADL and IADL using standardized tools, further examination of the relationship between functional daily care abilities touchscreen manipulation are necessary.

Future studies should examine the manipulation of touchscreens performed in less stable positions, such as while standing or walking in comparison to performance while sitting; this is important, since dual tasking, a common occurrence during the performance of many touchscreen tasks, has been shown to decline considerably in the elderly [42]. Kinematic and kinetic analyses of touchscreen use by older adults would also provide a more detailed and comprehensive understanding of the underlying issues in the operation of technological devices.

## 5. Conclusions

Older adults perform the more complex touchscreen manipulations more slowly and less accurately than middle-aged adults, regardless of their previous experience with smartphone manipulation. The TATOO-based evaluation of motor skill performance in touchscreen use demonstrates good usability of this instrument, indicating its usefulness as an assessment tool for older adults. TATOO may be an appropriate addition to the traditional clinical assessment tools to determine the ability of older adults to enhance their participation in technological activities and to provide tailor-made recommendations to adapt touchscreen devices. Finally, the current results have implications for developers and designers of touchscreen devices for older adults; there is a need to make special adjustments to these devices due to the decrease touchscreen manipulation ability of older adults (e.g., larger active area size, longer task duration).

## Figures and Tables

**Table 1:** Characteristics of the study samples (*n* = 53).

Variables	Middle-Age Group (*n* = 25)	Elderly Group (*n* = 28)
Age in years, mean ± SD (range)	53.4 ± 5.9 (46–63)	81.9 ± 4.2 (75–90)
Gender, *n* (%) Male/Female	7 (25)/21 (75)	6 (24)/19 (76)
Use of assistive device, *n* (%)	0 (0)	7 (25)

**Table 2:** Comparison of the Touchscreen Assessment Tool (TATOO) performance between age groups and with respect to hand dominance.

Task/Variable	Middle-Aged Group (*n* = 25) Dominant Nondominant		Elderly Group (*n* = 28) Dominant Nondominant		Group (*p*)	Hand (*p*)
1. Touch entire screen						
Reaction time (s)	1.9 ± 1.1	1.4 ± 0.8	2.6 ± 1.2	2.3 ± 1.5	0.0002	0.02
^#^ Taps	162.7 ± 37.7	153.4 ± 32.4	139.2 ± 32.2	114.0 ± 26.5	0.0007	<0.0001
2. Touch corners						
Reaction time (s)	1.2 ± 0.5	1.0 ± 0.4	2.0 ± 1.2	1.6 ± 0.7	<0.0001	NS
Test duration (s)	10.7 ± 0.8	10.8 ± 0.8	11.6 ± 1.1	11.4 ± 1.2	0.0001	NS
^#^ Taps	8.6 ± 0.9	8.4 ± 0.8	9.2 ± 1.9	10.0 ± 4.2	NS	NS
^#^ Correct Attempts	8.0 ± 0.0	8.0 ± 0.5	8.1 ± 0.7	9.0 ± 2.6	NS	NS
# Touches outside target	0.6 ± 0.9	0.3 ± 0.6	1.1 ± 1.8	0.8 ± 1.6	NS	NS
Flight time (s)	7.2 ± 0.7	7.5 ± 0.5	8.00 ± 0.9	10.6 ± 15.6	0.0002	NS
Touch time (s)	0.6 ± 0.3	0.7 ± 0.3	0.9 ± 0.6	1.8 ± 3.1	<0.0001	0.012
3. Double tap						
Reaction time (s)	1.1 ± 0.5	1.0 ± 0.3	1.8 ± 0.7	1.7 ± 0.7	<0.0001	NS
Test duration (s)	8.4 ± 1.5	8.3 ± 1.2	18.2 ± 10.3	20.1 ± 11.5	<0.0001	NS
^#^ taps	10.7 ± 2.4	10.4 ± 1.1	19.3 ± 12.0	22.4 ± 12.8	<0.0001	NS
^#^ Correct Attempts	10.0 ± 0.2	13.6 ± 18.0	9.1 ± 2.5	8.4 ± 3.0	0.03	NS
^#^ Touches outside target	0.6 ± 1.9	0.2 ± 1.0	1.9 ± 2.0	1.9 ± 6.0	0.0007	NS
Flight time (s)	5.3 ± 1.5	5.0 ± 1.1	13.7 ± 8.4	14.6 ± 9.4	<0.0001	NS
Touch time (s)	0.5 ± 0.2	0.7 ± 0.2	1.9 ± 2.0	4.3 ± 9.6	<0.0001	0.0004
4. Drag to different directions						
Reaction time (s)	2.0 ± 0.8	1.6 ± 0.5	4.5 ± 2.6	3.3 ± 2.2	<0.0001	0.00132
Test duration (s)	29.7 ± 15.0	25.9 ± 11.9	58.7 ± 24.0	59.0 ± 24.9	<0.0001	NS
^#^ Drag attempts	8.1 ± 3.4	6.2 ± 1.9	14.8 ± 9.4	11.1 ± 7.6	<0.0001	0.0002
^#^ Drags completed	4.9 ± 0.3	3.9 ± 1.6	3.8 ± 1.5	4.7 ± 0.6	NS	NS
^#^ Touches outside target	1.6 ± 1.6	2.7 ± 3.7	9.6 ± 11.5	14.0 ± 11.6	<0.0001	NS
Flight time (s)	12.5 ± 6.3	9.7 ± 6.8	27.1 ± 9.9	23.1 ± 9.7	<0.0001	0.013
Touch time (s)	13.7 ± 8.2	13.2 ± 5.8	19.4 ± 7.6	17.5 ± 7.6	0.0002	NS
5. Drag along a horizontal path						
Reaction time (s)	2.0 ± 1.4	1.0 ± 0.3	14.2 ± 50.8	2.8 ± 1.8	<0.000	<0.0001
Test duration (s)	13.0 ± 2.2	12.3 ± 3.7	19.6 ± 6.6	17.5 ± 4.5	<0.0001	NS
^#^ Drag attempts	5.1 ± 2.1	4.8 ± 2.0	5.7 ± 7.3	5.3 ± 2.7	NS	NS
^#^ Drags completed	3.0 ± 0	3.0 ± 0	3.0 ± 0	3.0 ± 0	NS	NS
^#^ Touches outside target	0.4 ± 0.7	0.1 ± 0.3	0.7 ± 1.4	1.0 ± 1.4	0.03	NS
Flight time (s)	5.5 ± 1.9	4.2 ± 1.7	8.9 ± 4.1	7.6 ± 3.8	0.04	NS
Touch time (s)	5.2 ± 1.2	5.9 ± 2.4	8.2 ± 4.1	7.4 ± 2.5	0.0001	NS
6. Pinch						
	1.6 ± 2.2	1.1 ± 0.4	2.6 ± 2.0	1.9 ± 1.6	0.0008	NS
	9.2 ± 6.8	9.9 ± 7.4	17.2 ± 12.5	12.6 ± 9.5	<0.0001	NS
	4.0 ± 9.1	4.2 ± 8.8	11.6 ± 24.4	3.44 ± 6.3	NS	NS
	4.2 ± 6.0	4.5 ± 4.4	10.0 ± 9.1	5.6 ± 4.2	<0.000	NS
	2.3 ± 1.7	2.8 ± 3.4	7.0 ± 14.5	4.4 ± 6.2	0.0006	NS

^#^ = number; s = seconds; NS = non-significant. * participants were all right-hand dominant.

**Table 3:** Means ± SDs for the System Usability Scale by item and group showing original and transformed scores.

	Middle-Aged (*n* = 25)		Elderly (*n* = 27)		*p* Value
Item	Original Score (1–5)	Transformed and Reversed Score (1–4)	Original Score (1–5)	Transformed and Reversed Score (1–4)	
1. I think that I would like to use this system frequently.	3.4 ± 1.76	2.4 ± 1.76	3.22 ± 1.69	2.22 ± 1.69	NS
2. I found the system unnecessarily complex.	1.28 ± 0.89	3.7 ± 0.89	1.48 ± 1.19	3.52 ± 1.19	NS
3. I thought the system was easy to use.	4.80 ± 0.50	3.8 ± 0.50	4.52 ± 0.98	3.52 ± 0.98	NS
4. I think that I would need the support of a technical person to be able to use this system.	1.92 ± 1.58	3.1 ± 1.58	2.15 ± 1.68	2.85 ± 1.68	NS
5. I found the various functions in this system were well integrated.	4.04 ± 1.37	3.0 ± 1.37	4.11 ± 1.28	3.11 ± 1.28	NS
6. I thought there was too much inconsistency in this system.	1.56 ± 1.04	3.4 ± 1.04	1.78 ± 1.37	3.22 ± 1.37	NS
7. I would imagine that most people would learn to use this system very quickly.	3.80 ± 1.68	2.8 ± 1.68	3.81 ± 1.57	2.81 ± 1.57	NS
8. I found the system very cumbersome to use.	1.32 ± 0.95	3.7 ± 0.95	1.37 ± 1.01	3.63 ± 1.01	NS
9. I felt very confident using the system.	4.56 ± 1.00	3.6 ± 1.00	4.51 ± 0.56	3.81 ± 0.56	NS
10. I needed to learn a lot of things before I could get going with this system.	1.40 ± 0.96	3.6 ± 0.96	1.63 ± 1.33	3.37 ± 1.33	NS
Total score		82.8 ± 13.7		80.2 ± 17.9	NS

SD = standard deviation.

## Data Availability

Data are available by direct contact with the corresponding author: michal.elboim@gmail.com and saadalexandra@gmail.com
